# A novel sleep aid device to reduce sleep latency using air–CO_2_ mixed gas

**DOI:** 10.3389/fneur.2023.1163904

**Published:** 2023-05-12

**Authors:** Hyewon Han, Dongsin Kim, Jin Seok Kim, Lee Ku Kwac, Janghun Hyeon, Junhyoung Oh

**Affiliations:** ^1^Department of Computer Engineering, Hongik University, Seoul, Republic of Korea; ^2^NYX Inc., Hanam, Republic of Korea; ^3^Department of Carbon Convergence Engineering, Jeonju University, Jeonju, Republic of Korea; ^4^Semiconductor Research Institute, Korea University, Seoul, Republic of Korea; ^5^Institute for Business Research and Education, Korea University, Seoul, Republic of Korea

**Keywords:** sleep-inducing, sleep latency, sleep disorder, sleep quality, sleep aid device

## Abstract

**Introduction:**

Sleep is an indispensable component of human life. However, in modern times, the number of people suffering from sleep disorders, such as insomnia and sleep deprivation, has increased significantly. Therefore, to alleviate the discomfort to the patient due to lack of sleep, sleeping pills and various sleep aids are being introduced and used. However, sleeping drugs are prescribed only to a limited extent due to the side effects posed by them and resistance to such drugs developed by patients in the long term, and the majority of sleep aids are scientifically groundless products. This study aimed to develop a device that induced sleep by spraying a mixed gas of carbon dioxide and air to create an environment that could induce sleep, similar to the inside of a sealed vehicle, to control oxygen saturation in the body.

**Methods:**

Based on the stipulated safety standards and the human tidal volume, the target concentration of carbon dioxide was determined to be of three types: 15,000, 20,000, and 25,000 ppm. After analyzing diverse structures for safely mixing gases, the most appropriate shape, the reserve tank, was selected as the best suited structure. Various variables, such as spraying angle and distance, flow rate, atmospheric temperature, and nozzle length, were comprehensively measured and tested. Furthermore based on this aspect, diffusion simulation of carbon dioxide concentration and actual experiments were conducted. To secure the stability and reliability of the developed product, an accredited test was performed to investigate the error rate of carbon dioxide concentration. Furthermore, clinical trials comprising polysomnography and questionnaires confirmed the effectiveness of the developed product not only in reducing sleep latency but also in enhancing the overall sleep quality.

**Results:**

When the developed device was put to use in reality, sleep latency was decreased by 29.01%, on average, for those with a sleep latency of 5 min or more, compared to when the device was not in use. Moreover, the total sleep time was increased by 29.19 min, WASO was decreased by 13.17%, and sleep efficiency was increased by 5.48%. We also affirmed that the ODI and 90% ODI did not decrease when the device was used. Although different questions may be raised about the safety of using a gas such as carbon dioxide (CO_2_), the result that tODI was not reduced shows that sleep aids using CO_2_ mixtures do not adversely affect human health.

**Discussion:**

The results of this study suggest a new method that can be used to treat sleep disorders including insomnia.

## 1. Introduction

Human beings spend about a third of their lives sleeping. Since sleep performs various functions, such as restoring physical and mental recovery, activating brain function, and promoting metabolism in a human being, it is closely related to modern society where human life expectancy has increased considerably. Sleep time is one of the crucial factors in life to the point where it is also related to the quality of life. However, sleep time of the Korean population remains at the lowest level among OECD countries (1), and the number of patients visiting hospitals in Korea is rapidly increasing due to sleep disorders represented by insomnia. Low sleep quality, especially the short duration of sleep, also increases suicidal thoughts in a person (2–4).

Sleep disorders include various conditions related to lack of sleep, such as failing to get a healthy sleep, unable to stay awake during waking hours despite having enough sleep, or suffering from physical and mental difficulties due to disturbed sleep cycles. Sleep disorders affect the depth of sleep and the quality of sleep time. Accumulated insufficient sleep causes extremely harmful and dangerous consequences related to health. A total of 30 to 48% of the adult population in the world suffer from sleep disorders, and 78% of them experience insomnia at least once a month (5–9). More than 680,000 patients are reported to visit hospitals for insomnia in Korea (10). Sleep is essential for maintaining human life, such as replenishing consumed energy, recovering from physical fatigue, and recovering from mental fatigue. Poor quality of sleep or sleep deprivation can cause problems related to physical activity and thereby increase the risk of exposure to various diseases. Hypertension, coronary artery disease, diabetes, and obesity are known as representative diseases that could arise due to lack of sleep (11, 12).

The existing products for sleep induction can be divided into two categories: medicines and sleep aid devices. The effectiveness of sleep induction has been proven by the use of sleeping medicines which are represented by sleeping pills and sleep inducers. However, a continuous drug use could trigger the risk of side effects and drug resistance. It has been reported that the users of hypnotic drugs have not only a three times higher mortality rate than non-users but also run a higher risk of cancer (13). In the case of temporary, situational, and short-term insomnia, drug use is helpful. Though, long-term prescriptions for chronic habit-forming patients are avoided to prevent drug resistance, dependence problems, and suicidal side effects caused by a drug overdose. Long-term drug treatment is not recommended for chronic habit-forming patients, as studies on the effectiveness and safety of long-term treatment are lacking, and the effect of treatment ceases when drug use is discontinued. In the case of existing sleep aid devices, they have been so formulated that they create a comfortable sleep environment rather than directly inducing sleep. Most of the sleep-inducing products provide only indirect effects, such as playing white noise or autonomous sensory meridian response (ASMR) sounds or using aroma scents that could help induce sleep. Products using brainwave resonance or magnetic fields have also been reported to cause only parasympathetic nerve activation, which does not lead to an actual decrease in sleep latency or improvement in sleep quality. Therefore, an effective novel method for inducing sleep that has no side effects and that can be used in the long term is needed.

In this study, we intend to develop a device that effectively shortens sleep latency by taking advantage of the fact that low oxygen saturation in the body causes drowsiness. To reduce oxygen saturation safely and efficiently, we used carbon dioxide. The inhalation of carbon dioxide is occasionally used as a therapy for the treatment of panic disorder by reducing anxiety. The usefulness of utilization of adequate utilization of carbon dioxide for sleep has already been demonstrated by the results of a previous research study. Choliz suggested that lowering the partial pressure of oxygen using carbon dioxide in insomnia patients reduces sleep latency (14). Ryan et al. reported that adding a little more carbon dioxide to the air improves sleep (15). Most of the products developed to improve sleep have been so far sold in the market without any evidence of effectiveness, thus an objective research to prove the effect of sleep-related products on sleep is required. Therefore, this study aimed to develop a new type of sleep aid device that applied the concept of control of oxygen saturation in the body to reduce sleep latency and to confirm the effect of shortening the sleep incubation period through clinical trials.

## 2. Materials and methods

### 2.1. CO_2_ concentrations suitable for inducing sleep

Carbon dioxide is a colorless, odorless, non-toxic gas. It is present in the atmosphere at an average concentration of approximately 400 ppm and is chemically stable and produced as a by-product of life's respiratory metabolism. In the United States of America, standards for exposure safety have been established through various experiments to cope with the problem of carbon dioxide concentration in confined spaces, such as submarines and spacecraft. Based on various experiments, the U.S. National Institute for Occupational Safety and Health (NIOSH) set a time-weighted average (TWA) of CO_2_ as less than 5,000 ppm for 8 h and a short-term exposure limit (STEL) as a maximum of 30,000 ppm within 15 min as safety standards (16). It has been confirmed that it is harmless to the human body within these concentrations. The Korean Ministry of Employment and Labor also stipulates the same concentration as NIOSH as a standard for exposure safety.

The breathing volume of a person depends on diverse variables, such as the body type and height, and it is normally calculated at the rate of 7 mL/kg for adults. The tidal volume measures around 300-500 mL/kg for average healthy adults. To increase the efficiency of sleep induction using carbon dioxide, an appropriate concentration of air–CO_2_ mixed gas should be sprayed depending on the amount of inhalation. The efficiency of sleep induction increases as sleep time decreases. To induce sleep in a short time, air with a higher concentration of carbon dioxide than that of the surrounding atmosphere must be inhaled. Therefore, we intend to spray an appropriate concentration of CO_2_ within the range of 15,000–25,000 ppm, so that induced sleep sets in a short time of fewer than 15 min. This range of CO_2_ concentrations is within the safety standard of 30,000 ppm of carbon dioxide concentration.

**Figure 1:**
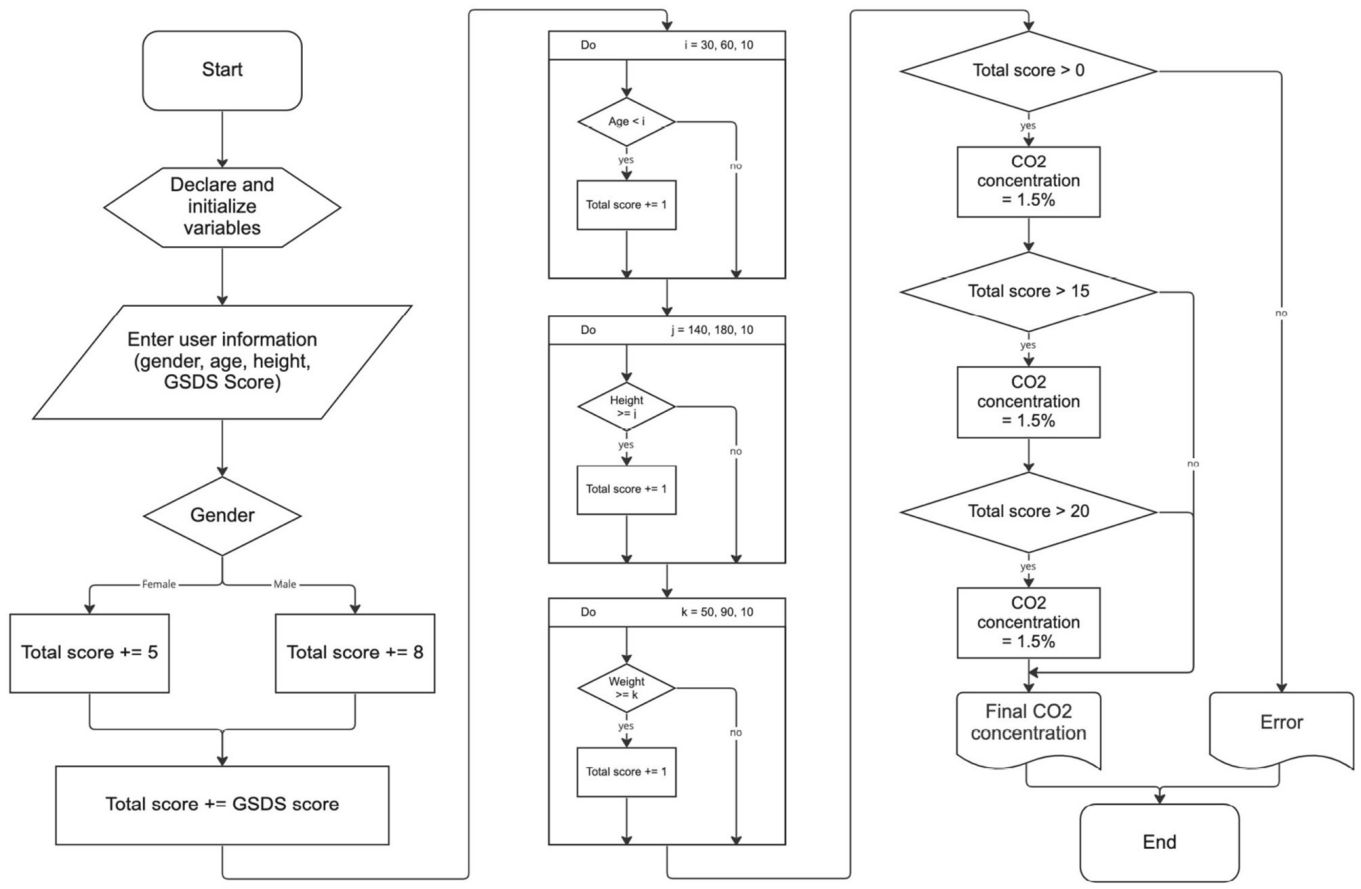
A flowchart of the algorithm for deciding CO_2_ concentration level of the mixed gas.

To provide optimized carbon dioxide concentrations to users, we developed and utilized an algorithm to estimate the user's tidal volume. The algorithm, which is shown as a flowchart in Figure 1, calculates a score that determines the carbon dioxide concentration level of the mixed gas based on the user's sex, age, height, weight, and the Minimal Insomnia Screening Scale. For the Minimal Insomnia Screening Scale, we used the General Sleep Disturbance Scale (GSDS). The scores calculated through the said algorithm were used to determine the concentration of carbon dioxide optimized for the user's physical condition among the lowest concentration of 15,000 ppm, intermediate concentration of 20,000 ppm, and the highest concentration of 25,000 ppm.

### 2.2. Controlling the concentration of air–CO_2_ mixed gas

#### 2.2.1. Diffusion of the air–CO_2_ mixed gas according to the spraying angle and distance

An experiment was conducted to study the effect of spraying angle and distance when the air–CO_2_ mixed gas was sprayed on the user's face. For the angle, three cases of 60°, 30°, and 0° were selected, and for the distance, two cases of 25 and 40 cm were selected. The flow velocity of the fan was stabilized by maintaining the voltage at 4.5 V, and the effect of spraying angle was confirmed at gas cylinder pressure concentrations of carbon dioxide between 18,000 and 20,000 ppm. In the case of CO_2_ measurement, a measurement device using 25 concentration sensors was manufactured in the form of 5-by-5 size, and the concentration corresponding to each sensor was measured and recorded.

The results of the experiment show that, the lower the angle and the longer the distance, the higher the pressure of carbon dioxide required for the target concentration. Therefore, the optimal angle for spraying carbon dioxide should range between 30° and 60° and the distance should be adjustable to the extent that the user does not feel uncomfortable. The experiment result, which is the measurement result of CO_2_ gas diffusion according to the spraying angle and distance, is shown in Table 1. The supply position of CO_2_ was fixed because it did not significantly affect gas diffusion.

**Table 1:** The measurement result of CO_2_ gas diffusion according to the spraying angle and distance.

**Angle (deg)**	**Length (cm)**	**Pressure (bar)**	**CO_2_ diffusion**
60	25	0.5	Stabilized
		40		0.65	
30	25	0.55	Highly concentrated
		40		0.75	
0	25	0.65	Unstable and asymmetric
		40		0.75	

#### 2.2.2. The fluid mixing method

Various techniques are available to create a uniform fluid flow. To select the most suitable method for our product, the methods, such as gas mixer, static mixer, venturi throat, and reserve tank, were selected and each technique was analyzed.

Gas mixer is used for medical or experimental applications that require precise control, and the fluid flow control valves that are suitable for implementing gas properties should be used to accurately mix the gas. Constant pressure is required to be maintained in the gas mixer, and a high level of technical skill is required to control and apply the flow rate of the fluid that is introduced from the fan since the device we intended to develop has to be operated by suctioning external air with the help of a fan.

Static mixer is a device in which a spiral axis is inserted into an existing flow path to mix the fluid, and it is mainly divided into horizontal and vertical parts according to the shape of a pipe. In the static mixer, the pressure loss increases with an increase in the number of spiral crossings, and it is necessary to consider the fan specification and the appropriate diameter of the pipe. To apply a static mixer to the concept design of our product, the inner diameter of the pipe should be manufactured within ϕ12 to ϕ20, which is relatively small to apply the method, and it may cause noise issues.

Venturi throat is a technique that uses a nozzle to rapidly increase the speed of the main fluid and lower the pressure to inhale the subfluid, and is used to mix another fluid of high pressure in the presence of a flow. It is usually employed in fields, such as boiler fuel supply and combustion, requires high pressure, and generates noise. Since the venturi throat is mainly used in large equipment, it is somewhat unreasonable to apply it to the concept design of our device. In addition, there is a disadvantage, in that it is difficult to control the subfluid pressure.

Reserve tank technique allows each of the two fluids to be drawn in and out of the same exhaust port. The design and technical level for the implementation are appropriate since the concept of technology lies in transforming and mixing the fluid flow. However, trapping two fluids in a reserve tank may cause spatial consumption due to creation of a place for mixing, and causing pressure loss. It is also greatly influenced by the tank shape.

The reserve tank method was selected in due consideration of the technical difficulty and design difficulties. The reserve tank was designed in two cases: A, which is based on the shape of a venturi tube, and B, which is based on a general spiral shape.

**Figure 2:**
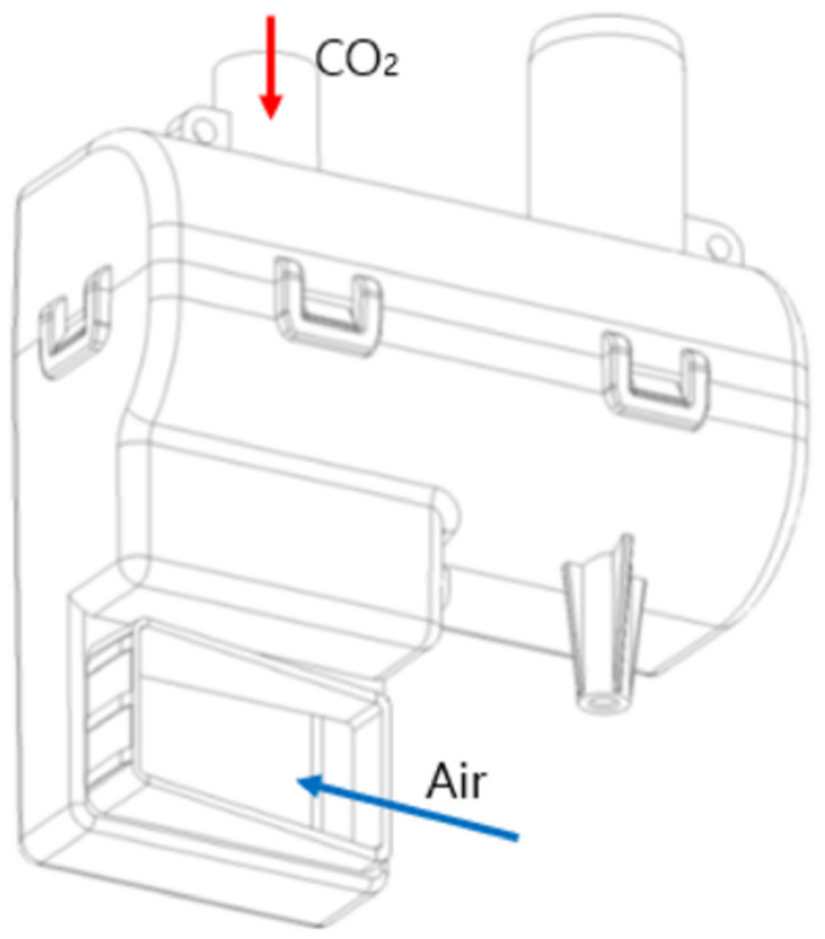
The final design for the reserve tank.

We conducted the flow analysis according to the designed structures A and B. From the analysis, we observed that fluids did not uniformly mix due to the fan wind pressure in A. Even in B, when the CO_2_ supply took place in the opposite direction to the supply flow path, fluids were not mixed uniformly. From the results of these two analyses, we found that when CO_2_ spray is used where air is injected, turbulence is naturally generated and the two fluids are mixed. Therefore, the final reserve tank was designed, as shown in Figure 2.

#### 2.2.3. Simulation of mixed gas diffusion according to temperature change

Diffusion simulations were conducted to confirm the effect of concentration distribution and flow velocity on the indoor temperature in summer and winter. For the simulation, we used COMSOL Multiphysics 5.5, which is a multiphysics simulation software. It was assumed that there was a head with a radius of 100 mm in an open space with a width of 2 m and a height of 1 m and the discharge port of the mixed gas was sprayed at a position of 250 mm from the head. In the case of flow velocity, 0.1 to 0.5 m/s was applied to set the range in which the user would not feel uncomfortable, and the analysis was performed in a time-dependent manner for 15 min.

Since the measurement unit of inlet in the simulation is mol per meter cubed (mol/m^3^), the unit of concentration is converted to parts per million (ppm). Considering that the molar volume of the ideal gas is 22.4 L and per meter cubed (m^3^) is 1,000 L, 1 mol/m^3^ is calculated to be 22,400 ppm. However, since the temperature correction is required to be carried out for gases, the same calculation process is applied to 24.45 and 23.88 L, which are the volumes of 25 and 18°C gases, respectively. Table 2 shows the ppm and percentage conversion results of target concentration at each temperature. A slight increase in concentration was observed when the temperature was lowered.

**Table 2:** The target concentration at each temperature in ppm and percentage.

**Temperature (°C)**	**mol/m^3^**	**ppm (%)**
25	1	24,450 (2.44)
		4.09	100,000 (10)
		0.82	20,000 (2)
18	1	23,880 (2.39)
		4.19	100,000 (10)
		0.84	20,000 (2)

Table 3 summarizes the concentration analysis results according to temperature and flow velocity. The concentration of the mixed gas decreased significantly at a relatively high temperature, and the concentration change increased at a lower flow velocity. However, no significant difference was found in each temperature during summer and winter. The flow velocity at the 0.1 m/s interval was also found to be insignificant.

**Table 3:** Concentration analysis results according to temperature and flow velocity.

**Temperature (°C)**	**Flow velocity (m/s)**	**Concentration (ppm)**	**Decreased concentration (ppm)**
18	0.5	23,800	76,200
		0.4	22,500	76,500
		0.3	22,200	76,800
		0.2	22,600	77,400
		0.1	21,700	78,300
25	0.5	23,200	76,800
		0.4	22,900	77,100
		0.3	22,600	77,400
		0.2	22,000	78,000
		0.1	21,200	78,800

### 2.3. Confirming mixed gas diffusion according to facial part distance

To secure the stability of gas concentration at the facial part, the concentration according to the distance was analyzed through a simulation by increasing it by 50 mm, i.e., from 150 to 450 mm. The flow velocity and temperature were fixed at 0.5 m/s and 25°C, respectively. By changing the discharged concentration of CO_2_ from 5 to 1 mol/m^3^, we attempted to find an appropriate concentration according to the distance.

Table 4 summarizes the CO_2_ concentration change according to the distance between the facial part and the discharge port, which is converted to part per million (ppm). Considering the error rate of simulation, a result of less than 25,000 ppm, which is lower than the safety standard of 30,000 ppm, was determined to be a valid result. It confirmed that the concentration of mixed gas decreased as the distance between the discharge port and the face increased.

**Table 4:** CO_2_ concentration change according to the distance between the facial part and the discharge port, which is converted to parts per million (ppm).

**Distance (mm)**	**Initial CO_2_ concentratlon (ppm)**	**Final CO_2_ concentratlon (ppm)**	**Decreased amount (ppm)**
150	50,000	37,500	12,500
		40,000	30,000	10,000
		30,000	22,500	7,500
		20,000	15,000	5,000
		10,000	7,500	2,500
200	50,000	34,200	15,800
		40,000	27,300	12,700
		30,000	20,500	9,500
		20,000	13,700	6,300
		10,000	6,900	3,100
250	50,000	31,600	18,400
		40,000	25,300	14,700
		30,000	19,000	11,000
		20,000	12,600	7,400
		10,000	6,300	3,700
300	50,000	30,500	19,500
		40,000	24,400	15,600
		30,000	18,300	11,700
		20,000	12,200	7,800
		10,000	6,100	3,900
350	50,000	29,200	20,800
		40,000	23,400	16,600
		30,000	17,600	12,400
		20,000	11,700	9,300
		10,000	5,800	4,200
400	50,000	29,000	21,000
		40,000	23,200	16,800
		30,000	17,400	12,600
		20,000	11,600	8,400
		10,000	5,800	4,200
450	50,000	31,600	21,800
		40,000	25,300	17,400
		30,000	19,000	13,000
		20,000	12,600	8,600
		10,000	6,300	4,300

**Figure 3:**
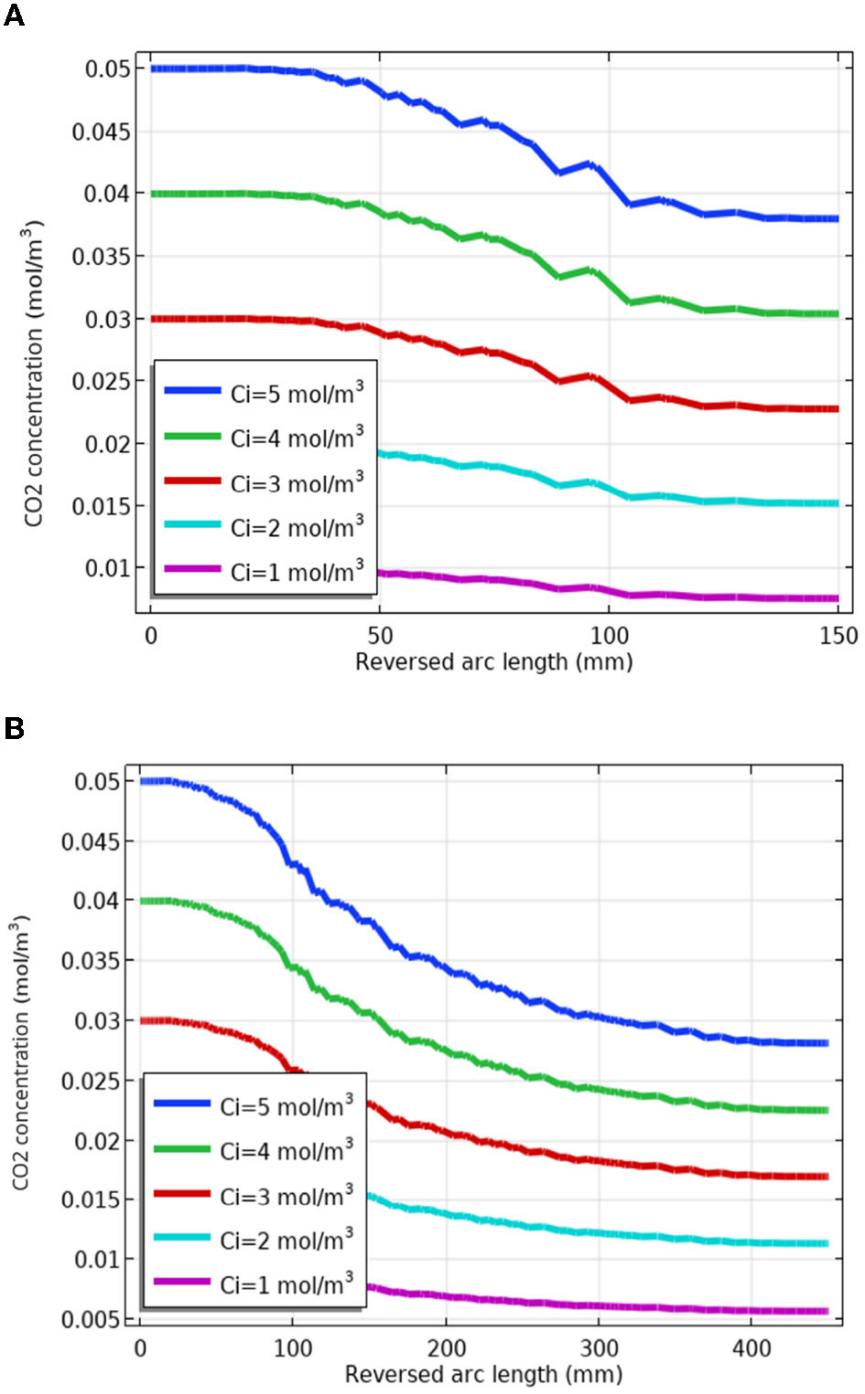
Changes in carbon dioxide CO_2_ concentration according to the distance between the facial part and the discharge port. **(A)** Change in CO_2_ concentration at 150 mm distance. **(B)** Change in CO_2_ concentration at 450 mm distance.

Figure 3A shows the concentration diffusion result according to the initial mol/m^3^ when the distance between the discharge port and the face is 150 mm, and Figure 3B shows the reduction result according to the initial mol/m^3^ when the distance is 450 mm.

### 2.4. Optimizing for a stable supply of the mixed gas

### 2.4.1. Voltage and pressure according to target CO_2_ concentration

To create the best sleep environment with the developed device, it is necessary to secure to supply mixed gases reliably, even if other variables, such as the use environment of the device and the concentration of carbon dioxide in the atmosphere, vary. Therefore, the three variables of fan speed through voltage control, CO_2_ supply pressure, and valve flow control were selected to secure optimal variables through actual experiments. The set range for each variable was 4 to 6 V for the voltage, 0.1 to 0.3 bar for CO_2_ input pressure, and 300 to 500 PWM for the valve flow rate.

**Figure 4:**
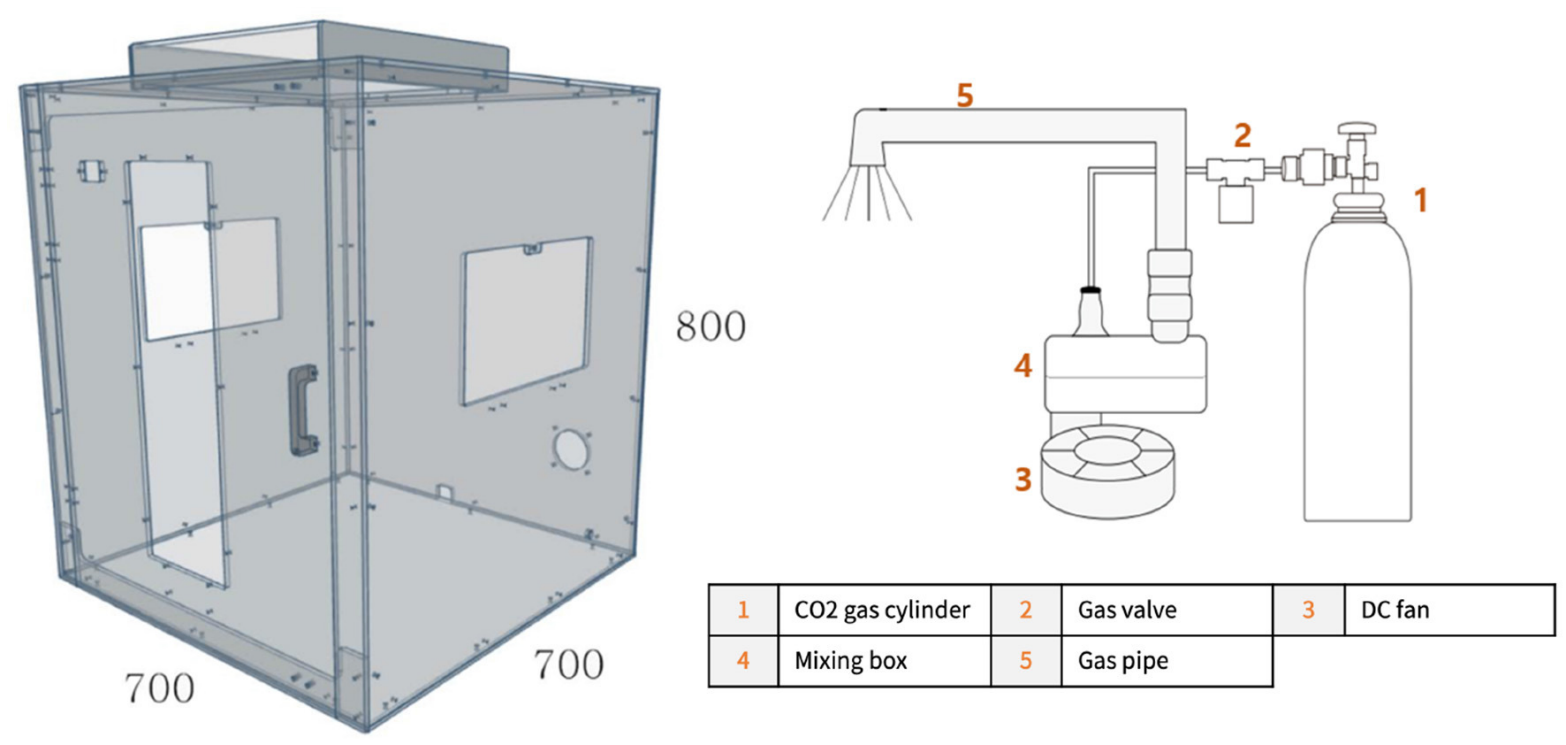
A schematic diagram of experimental environment and device.

The experiment was conducted with the implemented reserve tank in accordance with the final design of the reserve tank shown in Figure 2, a gas pipe, a CO_2_ cylinder, a valve, and a DC fan. The value of carbon dioxide concentration was measured by installing the CO_2_ sensor 25 cm below the fixed nozzle inside a chamber. Figure 4 shows the brief design of the chamber and experimental device. With the intention to stably mix the gas inside the tank, all variables were recorded at intervals of 2 s for 5 min after a stabilization time of 5 min. The values of each variable, such as the voltage of DC fan, CO_2_ input pressure, and flow rate of the valve, are displayed in Table 5, according to the target CO_2_ concentration value obtained through the experiment.

**Table 5:** The values of the voltage of DC fan, CO_2_ input pressure, and flow rate of the valve according to the target CO_2_ concentration value obtained through the experiment.

**CO_2_ concentration (ppm)**	**Voltage of DC fan (V)**	**CO_2_ input pressure (bar)**	**Flow rate of the valve (PWM)**
15,000	4	0.1	370
		5	0.2	450
		6	0.3	500
20,000	4	0.1	350
		5	0.2	380
		6	0.3	450
25,000	4	0.1	300
		5	0.2	370
		6	0.3	400

### 2.4.2. CO_2_ concentration change according to the nozzle design

To spray the mixed gas in a vertically downward direction, a gas-discharging nozzle with a bent end was designed. We aimed at stable mixing of fluids through a flow stream.

For the purpose of preventing the inflow of fine dust from the discharged part and securing safety, the concentration depending on the presence of a mesh on the discharging port was measured in the same environment. However, it was determined that the mesh worked to our disadvantage by interfering with the injection of mixed gas, instead of creating a sleep-inducing environment.

**Figure 5:**
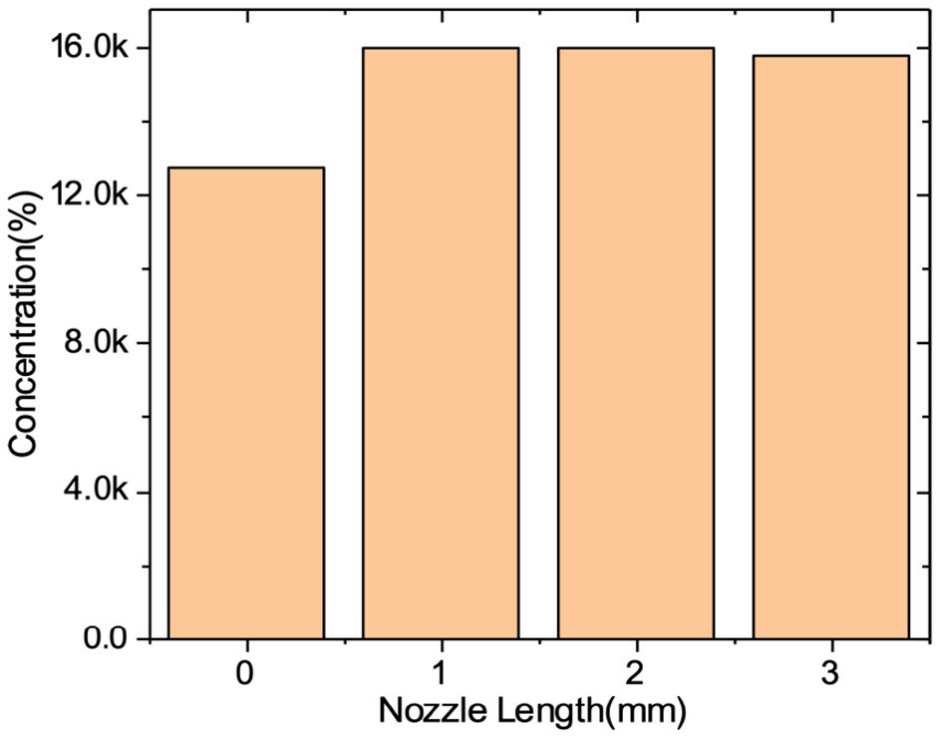
CO_2_ concentration according to the nozzle length.

Additionally, to examine the effect of the nozzle length, the length of the nozzle was manufactured to a size of 0 to 3 cm, and the carbon dioxide concentration of the mixed gas at the lower 25cm according to the different nozzle lengths was measured in the same environment. The measured concentrations are shown in Figure 5. At 0 cm, the mixed gas was not sufficiently transferred to the measurement position, and there were no significant difference lengths greater than 1 cm. Considering the corresponding results, the length of the nozzle was optimized to be 1 cm.

## 3. Results

### 3.1. Stability test results

An experiment to verify the error rate of CO_2_ concentration from the discharging port was conducted to secure the stability of the developed sleep aid device. The test was accredited by the Korea Laboratory Accreditation Scheme (KOLAS) of the Korean Agency for Technology and Standards. The error test of the CO_2_ concentration from the discharging port was conducted with the aim of achieving within 8% of the average value. A non-dispersive infrared (NDIR) sensor was installed inside the spraying nozzle to measure the CO_2_ concentration of mixed gas immediately before the injection, and the measurement was performed at room temperature and general atmospheric conditions. The CO_2_ concentration error test results are shown in Table 6, and the concentration was measured at the end of the discharging port. All test results are achieved within 8% of the error range.

**Table 6:** CO_2_ concentration error test results.

**Time**	**CO_2_ concentration (ppm)**	**Average CO_2_ concentration (ppm)**	**Error rate (%)**
11:35:00	45,010	43595.5	3.14
11:35:30	43,900			0.69
11:36:00	44,000			0.91
11:36:30	44,090			1.12
11:37:00	43,910			0.72
11:37:30	43,450			0.33
11:38:00	43,260			0.78
11:38:30	43,070			1.22
11:39:00	43,070			1.22
11:39:30	43,070			1.22
11:40:00	42,720			2.05

The concentration was measured at the end of the discharging port.

Another experiment to inspect the error rate was conducted by measuring the carbon dioxide concentration at the facial part, which is 25 cm away from the tip of the discharge port. The CO_2_ concentration error test results are shown in Table 7 and the concentration was measured at 25 cm apart from the end of the discharging port. These test results were also achieved within 8% of the error range.

**Table 7:** CO_2_ concentration error test results.

**Time**	**CO_2_ concentration (ppm)**	**Average CO_2_ concentration (ppm)**	**Error rate (%)**
11:35:00	13,150	13090.9	0.45
11:35:30	13,250			1.2
11:36:00	13,220			0.98
11:36:30	13,220			0.98
11:37:00	12,990			0.78
11:37:30	13,020			0.54
11:38:00	12,990			0.78
11:38:30	13,320			1.72
11:39:00	12,960			1.01
11:39:30	12,920			1.32
11:40:00	12,960			1.01

The concentration was measured at 25 cm apart from the end of the discharging port.

### 3.2. Clinical trial results

After securing the stability of the carbon dioxide concentration through the above-mentioned experimental results, a clinical trial was performed to confirm the effect of the developed device in reducing sleep latency. This study conducted a secondary analysis of sleep data collected *via* the Samsung Medical Center and NYX Incorporation. The detailed methods of the clinical study that collected the data have already been published (17). Since the device was designed to help people sleep, we excluded subjects who already sleep well from this analysis. Therefore, we used data from a subgroup of participants that excluded people with a sleep latency of less than 1 min or a sleep efficiency of 95% or higher.

The clinical trial was designed as a single-group crossover study to control for exogenous variables, such as the first-night effects of polysomnography and differences in subject characteristics. The said trial was conducted as a double-blind, sham-controlled study to exclude placebo effects due to experimental treatment. The person who operated the polysomnography machine to administer the polysomnography test could not be blinded, but the subject and the sleep physician who interpreted the results were blinded.

**Figure 6:**
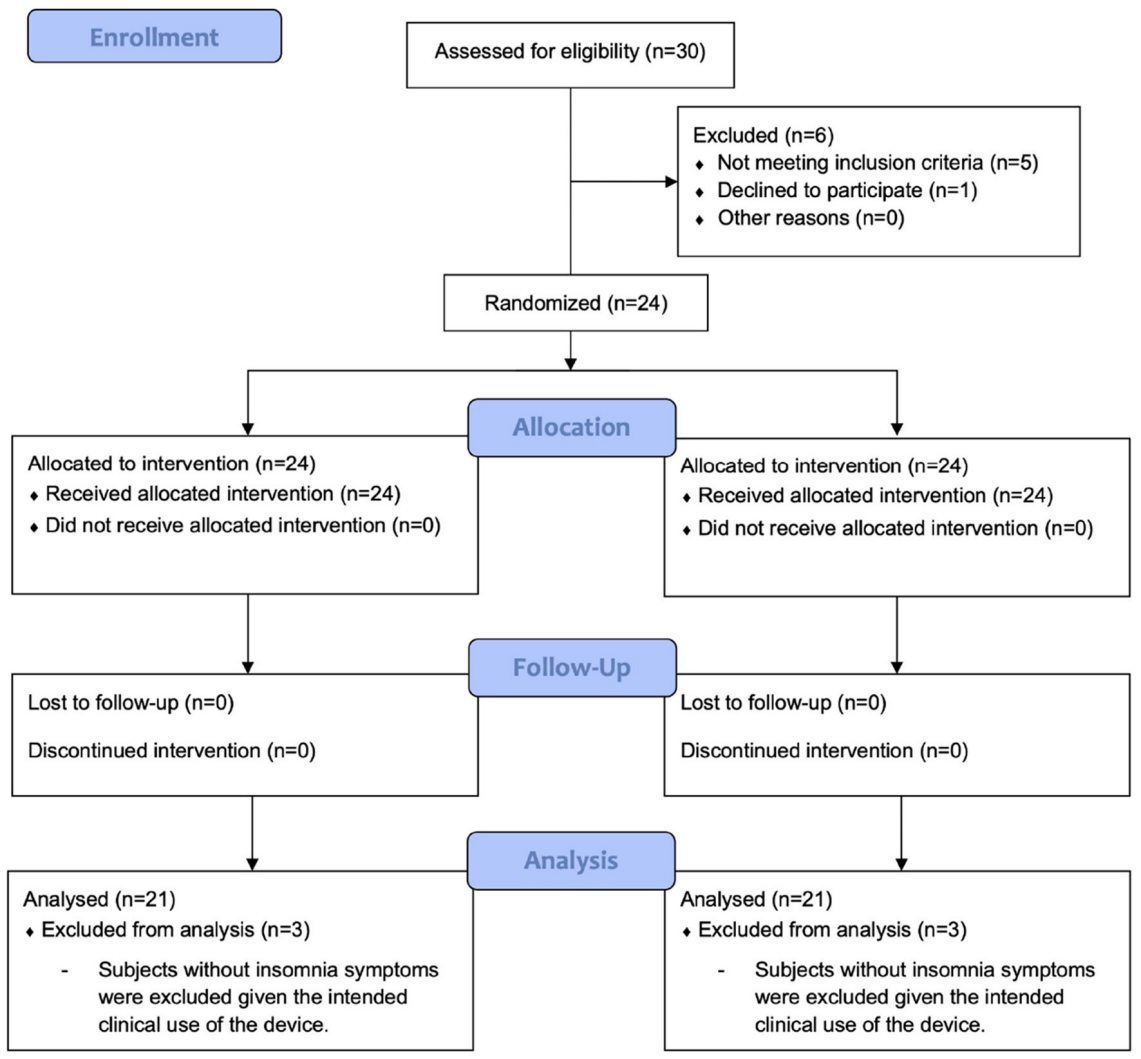
A flow diagram of this study showing the progression of the clinical trial according to CONSORT.

The flow diagram of this study displaying the progression of the clinical trial according to CONSORT is shown in Figure 6 (18). The polysomnography sessions were performed 14 days apart to prevent the learning effect on the examination and experimental treatment. The allocation order in the crossover design was 1:1, with a 50% chance of the first exposure to the mixed carbon dioxide. To exclude the effect of treatment order, the order of experimental and Siamese control treatments was determined by block randomization. The assignment order was performed by randomly selecting blocks with a block size of 6. Polysomnography was performed with an Embla N7000 (Medcare-Embla, Reykjavik, Iceland), and the results from the automated scoring system of the polysomnography equipment were used in this study. The study protocol was approved by the Institutional Review Board of the Samsung Medical Center (IRB No. 2021-04-133) and the entire study process was performed in accordance with the ethical standards of the Declaration of Helsinki. Written informed consent was obtained from each participant and all data were anonymized.

A total of 30 individuals were assessed for eligibility to participate in the study. Overall, 20- to 65-year-old adults with difficulty in sleep onset were selected as subjects for participation in the present study. Those patients diagnosed with cognitive decline, progressive mental or neurological diseases, lung disease, severe snoring, narcolepsy, REM sleep disorders, and clinically uncontrolled severe internal diseases, such as diabetes and hypertension, were excluded from participation in the experiment. Shift workers, pregnant women, lactating women, and those patients who were already diagnosed with insomnia were also excluded from participation in the experiment. Subjects were asked to record a sleep diary for 1 week during the screening phase. They were further excluded if they were deemed to have experienced sleep difficulties, based on a review of the sleep diary by a sleep physician with at least 20 years of experience in treating sleep patients. Clinical trials were conducted on a total of 24 subjects. However, as noted above, the device was designed to induce sleep in people with sleep difficulties, thus participants who were already sleeping well were further excluded from this analysis. We excluded three subjects who had a sleep latency of less than 1 min or a sleep efficiency of more than 95%, leaving 21 patients for analysis. For each subject, a Pittsburgh Sleep Quality Index (PSQI) score was obtained for a month. The subjects visited the Samsung Medical Center a total of four times, and they were examined with polysomnography and responded to the survey. A report on the descriptive statistics of demographic variables of participants is shown in Table 8.

**Table 8:** Descriptive statistics of demographic variables of participants.

**Demographic variable**	**Mean (SD)**	**Range**
Sex	Male: 8 (38.10%), Female: 13 (61.90%)	
Age (years)	49.24 (9.74)	29–61
Height (cm)	165.14 (9.76)	148–190
Weight (kg)	63.07 (13.76)	43–92
BMI (kg/m^2)	22.88 (2.80)	17.20–29

A paired *t*-test was performed to compare the trial results when the device was not used and when the device was used. The significance level was set at *p* < 0.05. Most of the comparison items showed a *p*-value less than 0.05, confirming that there was statistically significant improvement. The most principal variable, sleep latency, showed diverse results depending on the subject, but it was confirmed that the average decrease was 29.01% for those with more than 5 min of sleep latency. The trial results showed that the total sleep time was increased by 29.19 min, WASO was decreased by 13.17%, and sleep efficiency was increased by 5.48%. Moreover, the proportion of N1 sleep stage to total sleep time was decreased by 5.61% and the REM sleep to total sleep time was increased by 2.67%. As regards arousal, the total arousal index and the spontaneous arousal index were also decreased by 5.68 and 2.88%, respectively. These results indicate that the overall quality of sleep was improved when the device was in use. In the case of ODI, to statistically discover that the ODI did not significantly change when the device was used compared to when the device was not used, a paired *t*-test was conducted with the null hypothesis set to no decrease in the result and the alternative hypothesis set to decrease in the result. Both the ODI and 90% ODI had a *p*-value of 0.05 or above, confirming that the ODI did not notably decrease when using the device. Although the safety of carbon dioxide can be questioned, the result that the ODI was not reduced shows that sleep aids using CO_2_ mixed gas do not adversely affect human health. A statistical comparison of polysomnography results is summarized in Table 9. The results are indicated as mean (SD) [95% confidence interval].

**Table 9:** A statistical comparison of polysomnography results.

**Variable**	**Sleep without using the device**	**Sleep with using the device**	***p*-value**
Sleep latency (min)	9.86 (15.12) [3.39, 16.32]	8.24 (13.63) [2.41, 14.07]	0.037
TST (min)	283.55 (73.55) [252.09, 315.01]	322.56 (63.76) [295.29, 349.83]	0.000
WASO (%)	16.82 (17.24) [9.45, 24.20]	11.08 (8.59) [7.41, 14.75]	0.042
Sleep efficiency (%)	81.41 (17.89) [73.76, 89.06]	86.89 (10.36) [82.45, 91.32]	0.025
N1/TST (%)	20.69 (18.25) [12.88, 28.49]	15.08 (7.40) [11.91, 18.24]	0.041
REM/TST (%)	18.55 (6.13) [15.93, 21.17]	21.22 (4.32) [19.38, 23.07]	0.033
Total arousal index (/h)	24.03 (13.78) [18.13, 29.92]	18.35 (8.76) [14.60, 22.09]	0.003
Spontaneous arousal index (/h)	8.22 (8.08) [4.77, 11.68]	5.34 (3.24) [3.96, 6.73]	0.021
ODI (events/h)	7.64 (7.89) [4.26, 11.01]	9.52 (11.60) [4.56, 14.48]	0.827*
90% ODI (events/h)	0.77 (1.86) [-0.03, 1.56]	2.23 (6.66) [−0.62, 5.08]	0.841*

The results are indicated as mean (SD) [95% confidence interval]. TST, Total sleep time; WASO, wake after sleep onset; ODI, oxygen desaturation index; 90% ODI, 90% oxygen desaturation index (the count of ODI less than 90% per hour during sleep). ^*^indicates that the decrease in the result value that is not statistically significant, as shown by the *p*-value.

## 4. Discussion

Sleep is an essential part of life and has a huge impact on our daily lives, but in modern society, insomnia is prevalent, and sleep debt is increasing (19–21). Typical sleep-inducing methods include sleep drugs and sleep aid devices. However, it has been consistently pointed out that sleep drugs have side effects and drug resistance (22) in a patient, and sleep aid devices have been considered to bring about a number of disadvantages, in that they do not actually shorten sleep latency. Therefore, this study was conducted with the aim of developing a new type of sleep aid device that does not cause side effects or resistance and that has a direct sleep-inducing effect.

This study attempted to develop a device to induce sleep using oxygen saturation control in the body. We approached it by controlling the concentration of carbon dioxide in the atmosphere using a gas mixed with air and CO_2_. The total of four structures for making mixed gas, gas mixer, static mixer, venturi throat, and reserve tank, was analyzed for preparing the mixed gas, and the reservoir tank, which is the most appropriate form for the concept of the product to be developed, was adopted. In addition, various simulations were conducted to discover the degree of diffusion based on the design of the adopted mixed structure. Through experiments, the flow rate according to the voltage of the fan and the CO_2_ cylinder output pressure were determined, so that the carbon dioxide concentration in the facial part could achieve target values of 15,000, 20,000, and 25,000 ppm. To secure the stability of the discharge port, another experiment was also performed on the presence or absence of mesh and the length of the nozzle. According to the results of the experiment, it was decided not to use the mesh and the nozzle length was fixed at 1 cm.

Prototypes were developed with variables that were determined through the experiments, and the stability against carbon dioxide concentrations was secured through tests by a certified agency. Both the carbon dioxide concentration at the end of the discharge port and the concentration at the facial part, which is 25 cm away from the discharge port, were achieved within 8% of the target error value to prove the stability. Clinical trials were conducted at the Samsung Medical Center to prove the effect of substantial reduction on sleep latency. It was confirmed that there was a large deviation depending on the subjects, but the result of the clinical trials showed a reduction of up to 92% in sleep latency. The result of the clinical trials also confirmed that it was effective not only in reducing sleep latency but also in improving the overall sleep quality, including bringing about an improvement in aspects of the sleep stage, decrease in arousal, and increase in sleep efficiency. The device developed through this study induces sleep by spraying a gas mixture of carbon dioxide and air. But there are some challenges that arise, such as carbon dioxide, may cause situations leading to oxygen deprivation. From the data analysis, it was established that the ODI and 90% ODI did not decrease significantly when the device was used. From the fact that there are no substantial changes in the ODI when using this device compared to when not using it, we demonstrate that using the suggested device does not cause situations such as oxygen deprivation.

Among sleep disorders, especially insomnia, medication is recommended to be given to the patient only for a short period of time and only a minimum dose needs to be given to the patient to prevent misuse or side effects. Therefore, a non-drug treatment, such as cognitive behavioral therapy, is recommended. However, since there are only a few institutions that provide non-drug treatment in South Korea, not only is the accessibility to treatment low, but it also does not offer much scope that leads to give treatment for patients. For this reason, many attempts have been made to develop products that improve sleep and shorten the sleep latency period, but objective research is still lacking. Through this study, we have developed a sleep aid device that substantially reduces sleep latency and is effective in improving sleep quality. The results of the present research study suggest another option to treat insomnia without using hypnotic drugs that are at high risk for side effects.

## 5. Conclusions

In this study, a new type of sleep assistance device was developed that did not cause side effects or resistance and had a direct sleep-inducing effect in a patient. Air and carbon dioxide mixed gas was used to temporarily increase the concentration of carbon dioxide in the atmosphere to induce sleep. The target carbon dioxide concentration and the actual concentration of carbon dioxide after spraying were verified with the certified test agency to ensure stability of carbon dioxide concentration. Clinical trials have confirmed that it is not only effective in shortening sleep latency in a substance but also in improving the overall sleep quality.

## Data availability statement

The raw data supporting the conclusions of this article will be made available by the authors, without undue reservation.

## Ethics statement

The study protocol was approved by the Institutional Review Board of Samsung Medical Center (IRB No. 2021-04-133) and the entire process of the study was performed in accordance with the ethical standards of the Declaration of Helsinki. The patients/participants provided their written informed consent to participate in this study.

## Author contributions

HH wrote the first draft of the article. DK and JK performed the formal analysis and data curation. LK contributed to the design of the study. JH contributed to the conception of the study. JO supervised the overall research and administered the whole project. All authors contributed to the article and approved the submitted version.
